# ^1^H NMR and Multivariate Analysis for Geographic Characterization of Commercial Extra Virgin Olive Oil: A Possible Correlation with Climate Data

**DOI:** 10.3390/foods6110096

**Published:** 2017-11-07

**Authors:** Domenico Rongai, Nadia Sabatini, Laura Del Coco, Enzo Perri, Paolo Del Re, Nicola Simone, Donato Marchegiani, Francesco Paolo Fanizzi

**Affiliations:** 1 Consiglio per la ricerca in agricoltura e l’analisi dell’economia agraria (CREA), Research Centre for Plant Protection and Certification, via C.G. Bertero, 22, 00156 Roma, Italy; domenico.rongai@crea.gov.it; 2 Consiglio per la ricerca in agricoltura e l’analisi dell’economia agraria (CREA), Research Centre for Engineering and Agro-Food Processing, via L. Petruzzi, 75, 65013 Città Sant’Angelo, Italy; nadia.sabatini@crea.gov.it (Na.S.); paolo.delre@crea.gov.it (P.D.R.); nicola.simone@crea.gov.it (Ni.S.); donato.marchegiani@gmail.com (D.M.); 3 Dipartimento di Scienze e Tecnologie Biologiche ed Ambientali (Di.S.Te.B.A.), Università del Salento, Prov.Le Lecce-Monteroni, 73100 Lecce, Italy; laura.delcoco@unisalento.it; 4 Consiglio per la Ricerca in Agricoltura e l’Analisi dell’Economia Agraria-Centro di Ricerca per l’Olivicoltura e l’Industria Olearia, Contrada Li Rocchi, 87036 Rende, Italy; enzo.perri@entecra.it

**Keywords:** ^1^H NMR spectroscopy, EVOOs geographical origin, extra virgin olive oil, multivariate statistical analysis, historical climate data

## Abstract

^1^H Nuclear Magnetic Resonance (NMR) spectroscopy coupled with multivariate analysis has been applied in order to investigate metabolomic profiles of more than 200 extravirgin olive oils (EVOOs) collected in a period of over four years (2009–2012) from different geographic areas. In particular, commercially blended EVOO samples originating from different Italian regions (Tuscany, Sicily and Apulia), as well as European (Spain and Portugal) and non-European (Tunisia, Turkey, Chile and Australia) countries. Multivariate statistical analysis (Principal Component Analisys (PCA) and Orthogonal Partial Least Squares Discriminant Analysis (OPLS-DA)) applied on the NMR data revealed the existence of marked differences between Italian (in particular from Tuscany, Sicily and Apulia regions) and foreign (in particular Tunisian) EVOO samples. A possible correlation with available climate data has been also investigated. These results aim to develop a powerful NMR-based tool able to protect Italian olive oil productions.

## 1. Introduction

Olive oil is an ancient food widely spread throughout the Roman Empire. Olive trees are now mainly diffused around the Mediterranean Sea and Italy—located in the center of this region—is one of the most important olive producers. The extra virgin olive oil (EVOO) produced in various Italian regions has an excellent quality and is greatly appreciated worldwide for its attributes, which depend, among other factors, on the geographical origin. The importance of the geographical origin for EVOO has been documented by the European Union since 1992, when the Protected Designation of Origin (PDO) and Protected Geographical Indication (PGI) arrangements were created to protect and support foodstuffs of particular quality [1]. Bianchi et al. (1993) and Angerosa et al. (1999) used Isotopic Ratios Spectrometry (IRMS) to find olive oil components and to characterize the geographical origin [2,3]. The traceability and authenticity of olive oils have been widely investigated by Nuclear Magnetic Resonance (NMR) spectroscopy [4,5,6], Fourier transform infrared (FT-IR) [7] and mass spectrometry (MS) [6]. More recently a new regulation (EU 29/2012) [8] has affirmed that “*Extra virgin olive oil and virgin olive oil shall bear a designation of origin on the labelling*” and that “*the designation of origin shall appear on the packaging or on the label attached to the packaging*”. This EU regulation is very important, because it recognizes that the characteristics of virgin olive oils are closely related to geographical origin and also with the agricultural practices and techniques used during the EVOO extraction procedure. Since the authenticity of foodstuff origin represents a very important issue for the clear definition of the concept of food quality [9,10], in recent years, various analytical techniques, in combination with multivariate statistical analysis (MVA) methods, have been applied for this purpose. Resonance intensities of triacylglycerols obtained by ^13^C NMR spectroscopy were used to classify olive oils from three production areas of the Puglia region. By applying linear discriminant analysis (LDA) and the leave-one-out cross-validation procedure, most of the oils used for this purpose were correctly assigned to their groups [11]. Different techniques, such as IRMS and ^1^H NMR, in combination with MVA, have successfully been used to discriminate Italian olive oils from Tunisian ones [12]. The authors declared that the high quality of Italian olive oils was essentially due to the high content of squalene and unsaturated fatty acids, when compared to the Tunisian samples. NMR spectroscopy is one of the most widely used analytical techniques, together with MS, and has shown great success in food analysis [10,13,14,15], as it is able to supply a complete view of the olive oil metabolic profile, giving qualitative and quantitative information on its compounds, particularly on minor ones. Among the minor components, phenolic compounds are very important, because they have been linked to the healthy properties of EVOO. Spanish EVOOs were analyzed by a ^1^H NMR standard pulse and by an experiment suppressing the main lipid signals, allowing the detection of minor component resonances. Usually, standard ^1^H NMR is used to determine high concentrations of oil components, whereas the multisuppression approach is useful for increasing the sensitivity of NMR, allowing the detection of minor components [16,17,18]. Nowadays, the whole ^1^H NMR metabolic profile of EVOO is considered strongly related to the geographic origin of the olive oil, as well as to its cultivar, maturation index, and/or technological factors [9,19]. In recent works on metabolomics, the combination of ^1^H NMR fingerprinting and multivariate analysis has been successfully applied to predict the geographic origin of olive oils from different Mediterranean regions [18,20,21]. In our work, the ^1^H NMR-based MVA approach has been used also to examine the historical meteorological parameters in order to obtain a model for olive oil geographical origin prediction. Most of the studies carried out in the past regarding the provenance of olive oils were performed with oils obtained from olive trees belonging to a single cultivar and produced in the same year. In the present study all oil samples were blends and were collected in different years. Therefore, the aim of this research is to apply a new approach able to better discriminate commercial olive oils from different geographic areas and to reduce the influence of the year [22]. The originality of this research is that we also considered climate parameters alongside the NMR and MVA techniques, with the final aim of developing a powerful tool for easily tracing Italian olive oil production.

## 2. Materials and Methods

### 2.1. Samples

A total of 235 commercially blended EVOO samples were collected over a period of four years (2009–2012), from different Italian regions (Tuscany, Sicily and Apulia), as well as European (Spain, Portugal) and non-European (Tunisia, Turkey, Chile, Australia) countries. All the samples were stored in sealed dark glass bottles at room temperature in the dark prior to laboratory analysis. A detailed description, including the Italian regions and/or country of origin, the different cultivar composition (blend type) and number of olive oil samples for each country, is summarized in Table 1. Moreover, indicative average climate data have been reported and tentatively correlated to the oil characteristics, essentially for ease of access. (Florence-Parentola-Tuscany, Bari-Apulia, Catania-Sicily, Seville-Spain, Lisbon-Portugal, Sydney-Australia, Santiago-Chile, Tunis-Tunisia, Istanbul-Turkey). Average precipitation (monthly cumulative rainfall) and temperature were calculated for each studied area over a four-year period (2009–2012).

### 2.2. Nuclear Magnetic Resonance Spectroscopy

For each NMR sample preparation, 20 mg of olive oil was exactly weighed, dissolved in a volume of 0.9 mL of deuterated chloroform (CDCl_3_), and transferred directly to a 5 mm NMR tube. All the ^1^H NMR spectra were recorded on a 499.84 MHz spectrometer, operating at 11.7 T (Varian NMR UNITY INOVA Narrow Bore, workstation UNIX-based Sun Microsystems, Varian NMR Instruments, Palo Alto, CA, USA). Experiments (pulse program s2pul) were run at 298.15 K, using a 12 ms pulse 56 db (90° flip angle), an acquisition time of 5.82 s (64 k data points) a spectral width of 5500 Hz (11 ppm) and 16 transients. Prior to Fourier transformation, the free induction decays (FIDs) were zero-filled to 128 k and a −0.15 Hz line-broadening factor was applied.

### 2.3. Multivariate Data Processing

The data were Fourier-transformed, and phase and baseline corrected with ACD/NMR software (Advanced Chemistry Development, ACD/Spectrus software, version 2016.1.1, Toronto, ON, Canada). Chemical shifts were expressed in δ values relative to CHCl_3_ (δ 7.27 ppm) as internal reference. Spectra were segmented with a variable size intelligent bucketing width of 0.04 ppm and 50% looseness factor. The interval containing the signals of the solvent (in the range 7.60–6.90 ppm) was removed, and the sum of the remaining integrals (buckets) normalized for each spectrum. A total of 221 variables for each ^1^H NMR spectrum was obtained and considered for statistical analysis. Since the NMR spectra were dominated by the resonances of functional groups of all the fatty acids, each bucket row represents the entire NMR spectrum, and all the molecules present in the sample. The data table generated by all aligned bucket row reduced spectra was used for multivariate data analysis. The Pareto scaling method, which is performed by dividing the mean-centered data by the square root of the standard deviation, was then applied to the variables. Multivariate statistical analysis and graphics were obtained using SIMCA-P (version 14, Sartorius Stedim Biotech, Umea, Sweden). For multivariate statistical analyses of bucket reduced NMR spectra, different statistical procedures (principal component analysis (PCA) and orthogonal partial least squares discriminant analysis (OPLS-DA)) were used. PCA is used as a preliminary step in multivariate analysis of data. It works by reducing the dimensionality of data and reveals the presence of correlations among the samples. The principal components (PC*1*, PC*2*,…, PC*n*) are linear combinations of the original variables (in this case the NMR data) accounting for most of the variation in the data set. Hence, when a significant correlation occurs the number of useful PCs is much less than the number of original variables [23,24,25]. While PLS-DA is one of the most recent supervised MVA techniques used to discriminate samples with different characteristics according to known classification classes (such as cultivars and/or geographical origin) [22,26], we preferred OPLS-DA in our studies. As shown in several studies of metabolomics, OPLS-DA is a modification of the usual PLS-DA method that filters out variation that is not directly related to the focused discriminating response, by separating the portion of the variance useful for predictive purposes from the non-predictive variance (which is made orthogonal). The result is a model with improved interpretability. Furthermore, OPLS-DA condenses the predictive information into one component, facilitating the interpretation of spectral data. The R2(cum) and Q2(cum) are the two parameters used to describe the goodness of the model at the minimum number of components cumulatively required (cum) to optimally give account of the data variability. The R2 explains the total variations in the data, giving a quantitative measure of the goodness of fit. The goodness of prediction was estimated by Q2(cum), according to cross validation (sevenfold cross-validation) [26,27,28].

### 2.4. Chemicals

All chemical reagents for analysis were of analytical grade. Deuterated solvent (CDCl_3_ 99.8 atom%D) was purchased from was purchased from Sigma-Aldrich S.r.l. (Milan, Italy).

## 3. Results and Discussion

### NMR Spectroscopy and MVA

**Figure 1:**
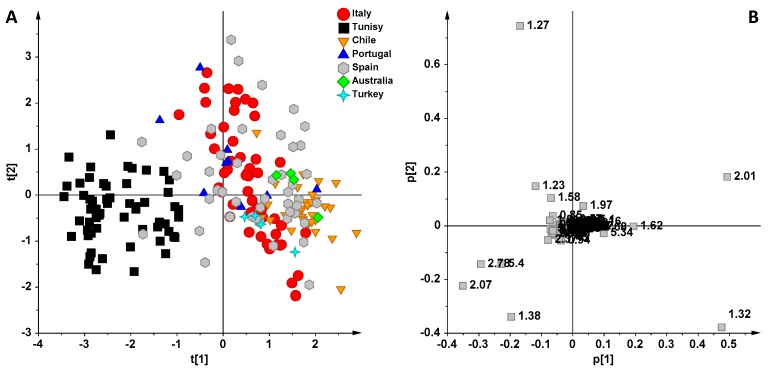
(**A**) Principal Component Analysis (PCA) (t[1]/t[2] scoreplot for the whole Nuclear Magnetic Resonance (NMR) dataset of extravirgin olive oils (EVOOs) (six components give R2X(cum) = 0.925 and Q2(cum) = 0.822). (**B**) Loading plot for the model; the variables indicated ppm in the ^1^H NMR spectra.

Multivariate statistical analysis was applied to all of the NMR data, focusing on the possible differences existing between Italian (from Tuscany, Sicily and Apulia regions) and foreign (Spain, Portugal, Tunisia, Turkey, Chile and Australia) EVOO samples. The fatty acid composition, together with squalene and β-sitosterol, was calculated by methods presented previously in the literature [29], on the basis of ^1^H NMR data. One-way analysis of variance (ANOVA) was performed to assess whether the means were significantly different among groups (see Tables S1 and S2). The obtained data indicated that all the investigated oils had fatty acid composition within the expected range for, in particular with regard to the polyunsaturated fatty acids (linolenic and linoleic fatty acids) and oleic acid. A first level of investigation was performed using the unsupervised exploratory statistical technique (PCA), without considering the climatic data, to look for trends among samples and/or possible outliers, which were excluded from further analyses, and to obtain a general overview of EVOOs (Figure 1). Of the original 221 variables per spectrum, six PCs were enough to describe 92.5% of the variance of the entire NMR dataset, giving R2X(cum) = 0.925 and Q2(cum) = 0.822 (with PC1, PC2 and PC3 describing 57.9%, 20.8% and 5.6% of the variance, respectively). The first principal component, PC1, gave a clear separation of Tunisian samples from the remaining classes, while all the other oils appeared to overlap considerably in the PC1/PC2 scoreplot (Figure 1A). Nevertheless, a certain degree of separation was also observed for Chilean oils, in particular on the third component (PC3), while European oils (Spanish, Portuguese, Italian) overlapped considerably in the scoreplot. By examining the loadings of the original variables it was possible to define the molecular components responsible for the observed trend (Figure 1B). In particular, Tunisian oils were characterized by a high relative content of polyunsaturated fatty acids, such as linolenic acid (1.38 ppm methylene protons of the unsaturated acyl groups, 2.78 ppm diallilyc groups, 5.40 ppm linolenic olefinic protons), while a high relative content of monounsatured fatty acids (1.32 ppm acyl group of oleic acid) was associated with all other oils.

**Figure 2:**
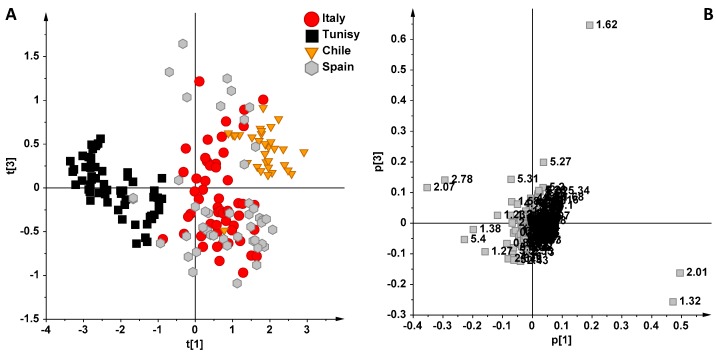
(**A**) PCA (t[1]/t[3] scoreplot for the whole NMR dataset of EVOOs (six components give R2X(cum) = 0.939 and Q2(cum) = 0.859). (**B**) Loading plot for the model; the variables indicated ppm in the ^1^H NMR spectra.

PCA analysis was further used considering only the EVOOs with a higher sample size, in order to increase the statistical significance for each class. Three oil groups were therefore excluded (with 4, 14, and 4 samples from Australia, Portugal, and Turkey, respectively) and the PCA was repeated using the remaining four clusters (with 61, 58, 34, and 62 samples from Italy, Tunisia, Chile, Spain, respectively). Analyzing the resulting PCA model (Figure 2, 93.9% of the variance with six PCs, R2X(cum) = 0.939 and Q2(cum) = 0.859), it could be observed again that the Tunisian samples were clearly separated on the first principal component PC1, while Chilean oils appeared clearly distinct from Italian and Spanish EVOOs, in particular when the third component (PC3) was considered. As described above, Tunisian oils were characterized by a high relative content of linolenic acid, while a high relative content of monounsatured fatty acids (1.32 ppm signal corresponding to the acyl group of oleic acid) was associated with the other oils. The most scattered clusters, Italian and Spanish oils, remained considerably overlapped in this PCA model, suggesting that further considerations are needed.

**Figure 3:**
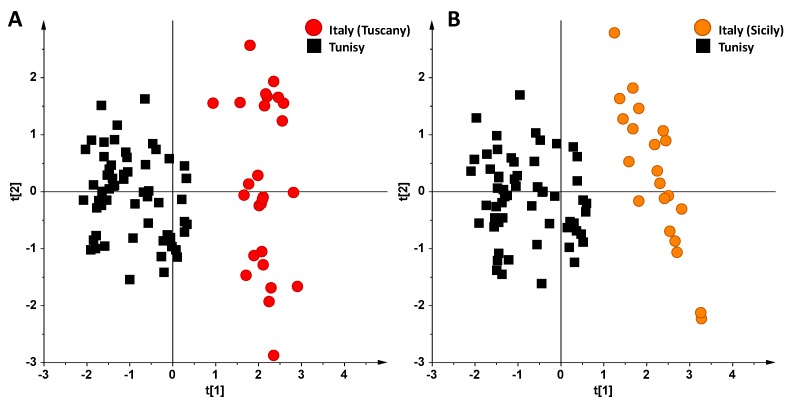
PCA scoreplots for EVOOs from Tuscan (**A**) and Sicilian (**B**) Italian regions vs. Tunisian oils.

**Figure 4:**
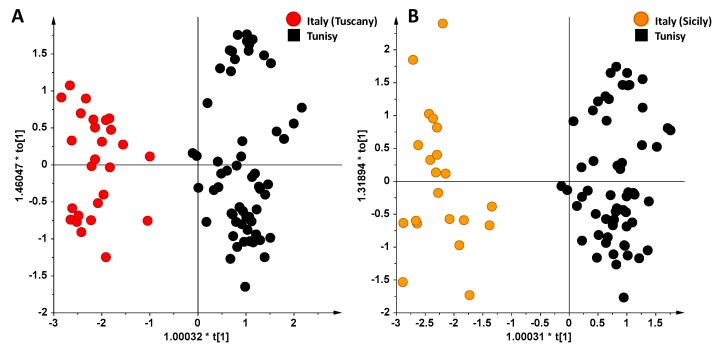
Orthogonal Partial Least Squares Discriminant Analysis, (OPLS-DA) scoreplots for EVOOs from Tuscan (**A**) and Sicilian (**B**) Italian regions vs. Tunisian oils.

In the first place, the unsupervised PCA and supervised OPLS-DA analyses were applied in order to deeply analyze the differences existing between Italian and Tunisian EVOO samples. Indeed, due to the recent introduction of Tunisian product in the EU olive oil market, and the serious impact on especially Italian production [30], differentiation of Tunisian EVOO appears to be a key issue [31]. In particular, taking into account the very different pedoclimatic conditions of the three Italian regions studied (Tuscany, Sicily and Apulia), sub-groups of samples from these regions were considered separately. Analyzing the resulting PCA scoreplots of Tunisian vs. Tuscan and Tunisian vs. Sicilian samples (Figure 3A,B respectively), a very good separation between the clusters was interestingly found even in the unsupervised analysis. Moreover, also in the case of Apulian vs. Tunisian oils, despite the low number of Apulian samples considered in this work, a good separation between the two clusters was observed, as already reported in other studies [32]. The samples were then analyzed by OPLS-DA in order to accurately analyze the differences observed in the PCA analysis and to investigate the goodness of fit (R2X) and prediction (Q2) for the models. In both cases (Tunisian vs. Tuscan and Tunisian vs. Sicilian samples), good OPLS-DA models were obtained, in which one predictive and two orthogonal components (1 + 2) gave R2X = 0.86, R2Y = 0.91 and Q2 = 0.89 and R2X = 0.85, R2Y = 0.92 and Q2 = 0.905, respectively. By considering the Q2 predictivity parameter for the OPLS-DA models of Figure 4A,B, it should be noted that both the OPLS-DA models showed a very high prediction ability (Q2 = 0.89 and Q2 = 0.905, respectively). Again, Tunisian oils were characterized by a high relative content of polyunsaturated fatty acids, such as linolenic acid (1.38 ppm, methylene protons of the unsaturated acyl groups, 2.78 and 5.40 ppm, linolenic diallilyc and olefinic protons, respectively), while a high relative content of oleic acid (5.34, 2.01, 1.32 ppm) was associated to both Tuscan and Sicilian oils.

**Figure 5:**
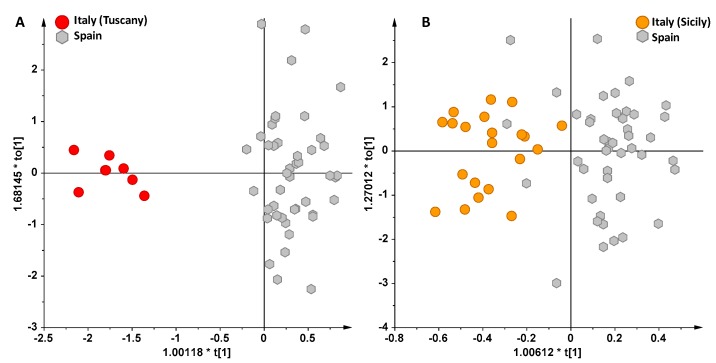
(**A**) OPLS-DA scoreplot for Italian (from Arezzo province, Tuscany region) and Spanish oils (1 + 4 + 0 components gave R2X = 0.91, R2Y = 0.89 and Q2 = 0.84). (**B**) OPLS-DA scoreplot for Italian (from Sicily region) and Spanish oils (1 + 6 + 0 components gave R2X = 0.9, R2Y = 0.71 and Q2 = 0.41).

Finally, analyzing the resulting OPLS-DA models between Italian (Sicilian, Tuscan) and Spanish EVOO samples, a good separation was obtained for Tuscan (in particular from Arezzo province, Tuscany region) vs. Spanish (Figure 5A) and for Sicilian vs. Spanish oils (Figure 5B). Again, also in the case of the Apulian (limited samples) vs. Spanish oils, a reasonable separation between the two clusters was observed, which is in agreement with results already reported in other studies [32].

Further consideration deserves to be given to the comparison of the OPLS-DA discriminating models and the average cumulative rainfall (mm) temperature (°C) data for the considered classes. A careful analysis of Table 1 data and the quality model descriptors for the OPLS-DA discriminations does not seem to give an indication of a clear correlation between both average rainfall and temperature differences and model discrimination performance (predictivity). Higher differences in average rainfall (Tuscan vs. Tunisian and Tuscan vs. Spanish oils) are generally associated with a more constant discriminating ability of the studied OPLS-DA models. This trend is also observed when considering average temperature differences. On the other hand, average country or regional temperature, although calculated for a wide time span (between the year 2009 and 2012), may not correctly account for the specific pedoclimatic conditions associated with the examined samples. Therefore conclusive correlations could not be obtained by using these simple climate descriptors, which were observed and calculated on a country and/or regional basis and chosen for the ease of their availability. Further studies possibly based on a detailed climate data detection and analysis are required in order to obtain sound correlation with specific EVOOs metabolic profiles.

## 4. Conclusions

NMR spectroscopy, combined with multivariate analysis techniques, successfully allowed characterization of metabolomic profiles of EVOOs, and their linkage with main cultivar composition and/or country of origin. Moreover, the fine composition of olive oil, and therefore its sensory characteristics, besides being strongly dependent on the nature of the cultivar used for its production, is also influenced by several other factors, like climate (temperature, relative humidity of summer months, yearly rainfall) as well as agricultural practices and technological factors (crop year, oil extraction system and storage time). In this work, the ^1^H NMR-based MVA approach was used on EVOOs collected over a four-year period (2009–2012), considering the potential influence of the historical meteorological parameter averages. Marked differences existing between Italian and foreign EVOO samples were observed, in particular when the three studied Italian regions (Tuscany, Sicily and Apulia), were considered separately. Higher differences in average rainfall and temperature generally resulted associated with a more constant discriminating ability of the studied OPLS-DA models. However, conclusive correlations could not be obtained by using the simple climate descriptors here considered, and further studies are required in order to obtain sound correlation of detailed climate parameters with specific EVOO metabolic profiles. It should be noted that the simplified approach used which takes into account indicative average weather data (rather than specific climate data variation across each country), was chosen for the ease of average climate data availability for each country. Nevertheless, these results suggest the possible use of NMR-based metabolic profiling for olive oils geographical origin prediction and assessment of the possible correlation with climate data. In fact, among the EVOOs studied here, the Tunisian case appears to be an interesting key issue, due to the recent introduction of Tunisian olive oil into the EU market. In this respect, it should be pointed out that the present oil characterization study is only aimed at clearly indicating possible metabolic profile differences among oils, rather than quality ranking.

## Figures and Tables

**Table 1:** Origins (from Italian regions and/or country), blend type and number of olive oil samples are reported in columns. Average of monthly cumulative rainfall (mm) and temperature (°C) are reported for each country and calculated over a four-year period (2009–2012).

Country (Region)	Cultivars	Number of Samples	Average Monthly Cumulative Rainfall (mm)	Average Temperature (°C)
Tuscany (Italy)	*frantoio, moraiolo, correggiolo, leccino, pendolino*	29	76.0	14.6
Apulia (Italy)	*coratina, ogliarola, nociara, leccino, peranzana*	9	48.8	15.7
Sicily (Italy)	*moresca, verrese, nocellara del belice, cerasuola, biancolilla, nocellara etnea*	23	45.6	17.3
Australia	*barnea, frantoio, correggiola*	4	45.4	21.7
Chile	*arbequina, leccino, bosana koroneiki, picua*	32	48.7	8.4
Portugal	*madural, cordovil, negrucha*	14	84.4	15.6
Spain	*picual, hojiblanca, arbequina, blanqueta, picudo*	62	50.3	14.0
Turkey	*sari ulak, ayvalik, beylik*	4	53.8	12.1
Tunisia	*chemlali, chetoui, chemchali*	58	22.2	20.7

Values of average of monthly cumulative rainfall (mm) and temperature (°C) are cited from Climate Change Knowledge Portal (http://sdwebx.worldbank.org/climateportal/).

## Supplementary Materials

The following are available online at http://www.mdpi.com/2304-8158/6/11/96/s1, Table S1: Fatty acid composition data calculated on the basis of ^1^H NMR, expressed in molar percentage. Table S2: One-Way ANOVA.

## References

[1] Regulation Regulation 2081/92/EEC. http://eur-lex.europa.eu/legal-content/EN/TXT/?uri=celex:31992R2081.

[2] Bianchi G., Angerosa F., Camera L., Reniero F., Anglani C. (1993). Stable carbon-isotope ratios (carbon-13/carbon-12) of olive oil components. J. Agric. Food Chem..

[3] Angerosa F., Breas O., Contento S., Guillou C., Reniero F., Sada E. (1999). Application of stable isotope ratio analysis to the characterization of the geographical origin of olive oils. J. Agric. Food Chem..

[4] Vlahov G., Shaw A.D., Kell D.B. (1999). Use of ^13^C nuclear magnetic resonance distortionless enhancement by polarization transfer pulse sequence and multivariate analysis to discriminate olive oil cultivars. J. Am. Oil Chem. Soc..

[5] Alonso-Salces R.M., Moreno-Rojas J.M., Holland M.V., Reniero F., Guillou C., Heberger K. (2010). Virgin olive oil authentication by multivariate analyses of ^1^H NMR fingerprints and δ^13^C and δ^2^H data. J. Agric. Food Chem..

[6] Cajka T., Riddellova K., Klimankova E., Cerna M., Pudil F., Hajslova J. (2010). Traceability of olive oil based on volatiles pattern and multivariate analysis. Food Chem..

[7] Bertran E., Blanco M., Coello J., Iturriaga H., Maspoch S., Montoliu I. (2000). Near infrared spectrometry and pattern recognition as screening methods for the authentication of virgin olive oils of very close geographical origins. J. Near Infrared Spectrosc..

[8] Commission Implementing Regulation (EU) No 29/2012. http://eur-lex.europa.eu/legal-content/EN/ALL/?uri=CELEX%3A32012R0029.

[9] Del Coco L., Mondelli D., Mezzapesa G.N., Miano T., De Pascali S.A., Girelli C.R., Fanizzi F.P. (2016). Protected Designation of Origin extra virgin olive oils assessment by nuclear magnetic resonance and multivariate statistical analysis:“Terra di Bari”, an Apulian (Southeast Italy) case study. J. Am. Oil Chem. Soc..

[10] Mannina L., Sobolev A.P., Capitani D. (2012). Applications of NMR metabolomics to the study of foodstuffs: Truffle, kiwifruit, lettuce, and sea bass. Electrophoresis.

[11] Vlahov G., Del Re P., Simone N. (2003). Determination of geographical origin of olive oils using ^13^C nuclear magnetic resonance spectroscopy. I—Classification of olive oils of the puglia region with denomination of protected origin. J. Agric. Food Chem..

[12] Camin F., Pavone A., Bontempo L., Wehrens R., Paolini M., Faberi A., Marianella R.M., Capitani D., Vista S., Mannina L. (2016). The use of IRMS, ^1^H NMR and chemical analysis to characterise Italian and imported Tunisian olive oils. Food Chem..

[13] Girelli C.R., De Pascali S.A., Del Coco L., Fanizzi F.P. (2016). Metabolic profile comparison of fruit juice from certified sweet cherry trees (*Prunus avium* L.) of ferrovia and giorgia cultivars: A preliminary study. Food Res. Int..

[14] De Pascali S.A., Coletta A., Del Coco L., Basile T., Gambacorta G., Fanizzi F.P. (2014). Viticultural practice and winemaking effects on metabolic profile of negroamaro. Food Chem..

[15] D’Imperio M., Mannina L., Capitani D., Bidet O., Rossi E., Bucarelli F.M., Quaglia G.B., Segre A. (2007). NMR and statistical study of olive oils from lazio: A geographical, ecological and agronomic characterization. Food Chem..

[16] Del Coco L., De Pascali S.A., Fanizzi F.P. (2014). ^1^H NMR spectroscopy and multivariate analysis of monovarietal EVOOs as a tool for modulating coratina-based blends. Foods.

[17] Ruiz-Aracama A., Goicoechea E., Guillen M.D. (2017). Direct study of minor extra-virgin olive oil components without any sample modification. ^1^H NMR multisupression experiment: A powerful tool. Food Chem..

[18] Longobardi F., Ventrella A., Napoli C., Humpfer E., Schutz B., Schafer H., Kontominas M.G., Sacco A. (2012). Classification of olive oils according to geographical origin by using ^1^H NMR fingerprinting combined with multivariate analysis. Food Chem..

[19] Del Coco L., De Pascali S.A., Fanizzi F.P. (2015). ^1^H NMR metabolic profiling of Apulian EVOOs: Fine pedoclimatic influences in salento cultivars. Royal Society of Chemistry.

[20] Mannina L., Sobolev A.P. (2011). High resolution NMR characterization of olive oils in terms of quality, authenticity and geographical origin. Magn. Reson. Chem..

[21] Rezzi S., Axelson D.E., Heberger K., Reniero F., Mariani C., Guillou C. (2005). Classification of olive oils using high throughput flow ^1^H NMR fingerprinting with principal component analysis, linear discriminant analysis and probabilistic neural networks. Anal. Chim. Acta.

[22] Girelli C.R., Del Coco L., Papadia P., De Pascali S.A., Fanizzi F.P. (2016). Harvest year effects on Apulian EVOOs evaluated by ^1^H NMR based metabolomics. PeerJ.

[23] Trygg J., Wold S. (2002). Orthogonal projections to latent structures (O-PLS). J. Chemom..

[24] Lindon J.C., Nicholson J.K., Holmes E. (2011). The Handbook of Metabonomics and Metabolomics.

[25] Miller J.N., Miller J.C. (2005). Statistics and Chemometrics for Analytical Chemistry.

[26] Girelli C.R., Del Coco L., Fanizzi F.P. (2015). ^1^H NMR spectroscopy and multivariate analysis as possible tool to assess cultivars, from specific geographical areas, in evoos. Eur. J. Lipid Sci. Technol..

[27] Westerhuis J.A., van Velzen E.J., Hoefsloot H.C., Smilde A.K. (2010). Multivariate paired data analysis: Multilevel PLSDA versus OPLSDA. Metabolomics.

[28] Trygg J. (2002). O2-PLS for qualitative and quantitative analysis in multivariate calibration. J. Chemom..

[29] Merchak N., El Bacha E., Khouzam R.B., Rizk T., Akoka S., Bejjani J. (2017). Geoclimatic, morphological, and temporal effects on lebanese olive oils composition and classification: A ^1^H NMR metabolomic study. Food Chem..

[30] Regulation (EU) 2016/580. http://www.mvo.nl/media/reg_2016_580_tunisian_olive_oil.pdf.

[31] Girelli C.R., Del Coco L., Fanizzi F.P. (2017). Tunisian extra virgin olive oil traceability in the EEC market: Tunisian/Italian (Coratina) EVOOs blend as a case study. Sustainability.

[32] Del Coco L., Schena F.P., Fanizzi F.P. (2012). ^1^H nuclear magnetic resonance study of olive oils commercially available as Italian products in the United States of America. Nutrients.

