# Supplementary material for: 1H NMR and Multivariate Analysis for Geographic Characterization of Commercial Extra Virgin Olive Oil: A Possible Correlation with Climate Data
Source: Foods. 2017 Nov 7;6(11):96. doi: 10.3390/foods6110096 (PMC5704140; doi:10.3390/foods6110096)
Supplement: Supplementary file 1 [file foods-06-00096-s001.pdf]

^{1}H NMR and Multivariate Analysis for Geographic Characterization of Commercial Extra Virgin Olive Oil: A Possible Correlation with Climate Data

Table S1. Fatty acid composition data calculated on the basis of ^{1}H NMR, expressed in molar percentage.

Region/Country	Linolenic Acid	Linoleic Acid	UFA	MUFA (Oleic Acid)	Squalene	β-Sitosterol
Toscana	0.78	7.94	73.37	64.66	2.05	0.27
Sicilia	0.71	9.14	74.32	64.46	2.05	0.23
Puglia	0.76	9.26	75.50	65.48	1.86	0.21
Tunisia	0.74	16.12	69.51	52.65	1.66	0.29
Chile	0.69	6.26	75.42	68.46	2.09	0.23
Portugal	0.73	8.27	73.36	64.36	1.85	0.31
Spagna	0.71	7.78	73.84	65.35	2.00	0.24
Australia	0.75	5.84	74.54	67.95	2.22	0.22
Turchia	0.70	9.47	72.64	62.47	2.33	0.30

UFA: unsaturated fatty acid. MUFA: monounsaturated fatty acid. Reference: Merchak, N.; El Bacha, E.; Khouzam, R.B.; Rizk, T.; Akoka, S.; Bejjani, J. Geoclimatic, morphological, and temporal effects on lebanese olive oils composition and classification: A ^{1}H NMR metabolomic study. *Food chemistry* **2017**, *217*, 379-388.

Table S2. One-Way ANOVA.

Attribute_Y	Attribute X	Description			Statistical Test		
		**Value**	**Average**	**Std-dev**	**Variance Decomposition**		
%Ln	Region	**Toscana**	0.7801	0.0527	**Source**	**Sum of square**	**d.f.**
		**Sicilia**	0.7113	0.0567	BSS	0.1483	8
		**Puglia**	0.7586	0.0625	WSS	0.8626	196
		**Tunisia**	0.7401	0.0449	TSS	1.0109	204
		**Chile**	0.6932	0.0674	**Significance Level**		
		**Portogallo**	0.7262	0.0785	**Statistics**	**Value**	**Proba**
		**Spagna**	0.7102	0.0933	Fisher's F	4.212873	0.000111
		**Australia**	0.7456	0.0665			
		**Turchia**	0.6989	0.0510			
		**Value**	**Average**	**Std-dev**	**Variance Decomposition**		
%L	Region	**Toscana**	7.9365	0.8026	**Source**	**Sum of Square**	**d.f.**
		**Sicilia**	9.1443	0.7493	BSS	3064.0098	8
		**Puglia**	9.2636	1.8809	WSS	1060.1774	196

		Value	Average	Std-dev
		**Tunisia**	16.1206	2.7524
		**Chile**	6.2647	1.3447
		**Portogallo**	8.2742	2.3938
		**Spagna**	7.7812	3.2854
		**Australia**	5.8446	1.0478
		**Turchia**	9.4721	1.0373

TSS	4124.1872	204
**Significance level**		
**Statistics**	**Value**	**Proba**
Fisher's F	70.807244	0.000000

%UFA	Region	**Value**	**Average**	**Std-dev**
		**Toscana**	73.3734	1.9277
		**Sicilia**	74.3158	2.2741
		**Puglia**	75.5020	1.0008
		**Tunisia**	69.5089	1.3819
		**Chile**	75.4207	1.7388
		**Portogallo**	73.3643	1.7448
		**Spagna**	73.8449	1.9564
		**Australia**	74.5445	3.0442
		**Turchia**	72.6362	2.2141

**Variance Decomposition**		
**Source**	**Sum of square**	**d.f.**
BSS	1011.8906	8
WSS	639.1924	196
TSS	1651.0830	204
**Significance Level**		
**Statistics**	**Value**	**Proba**
Fisher's F	38.785380	0.000000

%MUFA	Region	**Value**	**Average**	**Std-dev**
		**Toscana**	64.6567	2.3109
		**Sicilia**	64.4602	2.5256
		**Puglia**	65.4799	2.1788
		**Tunisia**	52.6481	3.8532
		**Chile**	68.4629	2.6334
		**Portogallo**	64.3639	3.8619
		**Spagna**	65.3535	4.8761
		**Australia**	67.9543	2.5815
		**Turchia**	62.4652	2.8104

**Variance Decomposition**		
**Source**	**Sum of square**	**d.f.**
BSS	7491.3733	8
WSS	2555.2227	196
TSS	10046.5959	204
**Significance Level**		
**Statistics**	**Value**	**Proba**
Fisher's F	71.828827	0.000000

%squalene	Region	**Value**	**Average**	**Std-dev**
		**Toscana**	2.0477	0.4621
		**Sicilia**	2.0452	0.2738
		**Puglia**	1.8570	0.2118
		**Tunisia**	1.6623	0.1368
		**Chile**	2.0939	0.3565

**Variance Decomposition**		
**Source**	**Sum of Square**	**d.f.**
BSS	6.9502	8
WSS	16.9565	196
TSS	23.9067	204
**Significance Level**		

		**Portogallo**	1.8540	0.1948	**Statistics**	**Value**	**Proba**
		**Spagna**	2.0019	0.2330	Fisher's F	10.042216	0.000000
		**Australia**	2.2202	0.7850			
		**Turchia**	2.3309	0.3408			
%b-sitosterol	Region	**Value**	**Average**	**Std-dev**	**Variance Decomposition**		
		**Toscana**	0.2664	0.0315	**Source**	**Sum of square**	**d.f.**
		**Sicilia**	0.2343	0.0575	BSS	0.1506	8
		**Puglia**	0.2139	0.0827	WSS	0.9355	196
		**Tunisia**	0.2878	0.0377	TSS	1.0861	204
		**Chile**	0.2328	0.1055	**Significance Level**		
		**Portogallo**	0.3083	0.1053	**Statistics**	**Value**	**Proba**
		**Spagna**	0.2369	0.0727	Fisher's F	3.944440	0.000239
		**Australia**	0.2241	0.1174			
		**Turchia**	0.2971	0.0841			
